# Review, Properties, and Synthesis of Single-Switch Non-Isolated DC-DC Converters with a Wide Conversion Range

**DOI:** 10.3390/s24072264

**Published:** 2024-04-02

**Authors:** Fernando Lessa Tofoli, Thaís Martins Jajah Carlos, Aniel Silva Morais

**Affiliations:** 1 Department of Electrical Engineering, Federal University of São João del-Rei, São João del-Rei 36307-352, Brazil; 2 Faculty of Electrical Engineering, Federal University of Uberlândia, Uberlândia 38408-100, Brazil; thaisjajah@gmail.com (T.M.J.C.); aniel@ufu.br (A.S.M.)

**Keywords:** non-isolated DC-DC converters, graft scheme, quadratic converters, wide conversion range

## Abstract

The cascaded connection of power converters extends conversion ranges but requires careful consideration due to high component count and efficiency concerns, as power is processed redundantly. Furthermore, using several active switches that must be turned on simultaneously may introduce significant drive and control complexity. To overcome this limitation, single-switch quadratic DC-DC converters have been proposed in the literature as a prominent choice for various applications, such as light-emitting diode (LED) drivers. Nevertheless, the motivation behind the conception of such topologies, beyond extending the conversion ratio, remains unclear. Another unexplored issue is the possibility of obtaining single-switch versions of cascaded converters consisting of multiple stages. In this context, this work investigates the synthesis of single-switch non-isolated DC-DC converters for achieving high step-down and/or high step-up based on the graft scheme. Key issues such as the voltage gain, additional stresses on the active switches, component count, and behavior of the input current and output stage current are addressed in detail. An in-depth discussion is presented to identify potential advantages and shortcomings of the resulting structures.

## 1. Introduction

DC-DC converters are crucial for modern applications involving electronic equipment. Typical examples include switch-mode power supplies (SMPSs), DC motor drives, renewable energy conversion systems, microgrids, light-emitting diode (LED) drivers in lighting systems, electric vehicles (EVs), uninterruptible power systems (UPSs), and electric aircraft, among others [1]. They play an important role in adapting DC voltage levels between the source and loads, as well as in controlling power flow effectively and maintaining the output voltage constant, particularly when the load power varies [2].

The classical non-isolated DC-DC converters, such as the buck, boost, buck–boost, Ćuk, single-ended primary-inductance converter (SEPIC), and Zeta topologies, are still used in many practical applications nowadays [3]. All of them derive from a basic arrangement referred to as the canonical cell or pulse-width modulation (PWM) switch [4]. They have also led to the conception of numerous derived structures with improved characteristics over the last decades [5]. However, they are limited when dealing with wide voltage conversion ranges, primarily because the ratio between the output voltage and the input voltage depends solely on the duty cycle associated with the active switch [6].

In turn, incorporating high-frequency transformers can extend the voltage conversion ratio of DC-DC converters [7]. The classical isolated topologies, such as the flyback, forward, push–pull, half-bridge, and full-bridge converters, allow for adjusting the output voltage according to not only the duty cycle but also the transformer turns ratio, aiming to achieve high step-up or high step-down [8]. Since high-frequency transformers rely on materials with high magnetic permeability, such as ferrite, unlike their low-frequency counterparts based on silicon–steel, they may be limited in size due to the lack of mechanical robustness and ability to process power levels up to 50 kW; this being a conservative estimate according to [9]. Nevertheless, the utilization of high-frequency transformers can significantly impact power density, especially in high-power applications.

Non-isolated DC-DC converters with a wide conversion range represent a prominent choice for applications in which galvanic isolation is not a mandatory requirement [10]. Perhaps the first work on this subject is [11], which proposed a modular voltage-divider Ćuk converter with multiple active switches connected to a common ground for achieving large voltage step-down ratios. However, the first study to present single-switch DC-DC converters with a wide conversion range seems to be [12], which introduced the so-called quadratic DC-DC buck, boost, and buck–boost converters, as well as other structures based on the combinations of distinct arrangements. In fact, such topologies consist of the cascade association of two conversion stages, yielding redundant power processing and low efficiency. However, the concept that allows for deriving single-switch structures from cascaded converters whose active switches share a common node was only formalized in [13] in terms of the graft scheme.

More recently, non-isolated high step-up DC-DC converters have received significant attention from researchers and experts in the field of power electronics, especially owing to the increasing penetration of grid-connected renewable energy conversion systems [14]. According to [15], one can extend the conversion ratio of the traditional boost converter using switched capacitors, switched inductors, voltage multipliers, voltage lifting techniques, magnetic coupling, and cascaded stages, or even consider a combination of such solutions. A plethora of topologies are available in the recent literature, but discussing them in detail falls outside the scope of this work.

Switched capacitors can achieve high efficiency at low power levels, but the resulting structures may have a limited voltage-boosting capability while requiring the association with other techniques to increase the voltage gain. This inconvenience can be overcome with the use of switched inductors but at the cost of increased dimensions and weight, as well as high electromagnetic interference (EMI) levels. Voltage multipliers are simple solutions for deriving high step-up converters based on a modular approach, although a high component count may be necessary, thus affecting overall power density.

Voltage lifting techniques offer flexibility and adaptability to various input and output configurations, but thermal management becomes a major concern, particularly at high power levels. Magnetic coupling approaches based on built-in transformers and coupled inductors allow for adjusting the voltage gain according to the turns ratio associated with the windings, but this may be a somewhat limited solution at high power levels owing to the increased dimensions of magnetic elements. Cascaded converters can also provide high step-up, but the high component count and sensitivity to parameter variations and mismatches associated with the converter stages may restrict them to low-power applications.

On the other hand, non-isolated high step-down DC-DC converters are available in a much smaller number of publications [16]. Possible strategies for extending the conversion ratio of the conventional buck converter include cascaded stages, coupled inductors, switched capacitors, and switched inductors [17].

Although cascaded and quadratic converters may lead to poor efficiency, as previously mentioned, several topologies with enhanced performance in terms of voltage gain, current/voltage stresses, and efficiency are available in recent works [18]. However, it is reasonable to state that the basic structures introduced in [12] are still an adequate choice for low-power applications involving intermediate voltage gains associated with moderate input voltage and/or output voltage levels, according to [19]. Thus, the authors in [20] formulate a systematic method for the synthesis of power converters based on a specified voltage conversion ratio using the quadratic buck–boost converter as an example. This inverse problem is also addressed in [21], where the conception of the quadratic buck topology is investigated from the forward and reverse perspectives. One can also derive quadratic converters using the fundamental flux balance equation across the inductors [22].

Some modern applications may benefit significantly from the utilization of quadratic converters. For instance, three quadratic DC-DC buck topologies that can be reconfigured in the form of semi-quadratic buck–boost arrangements are proposed in [23] for electrolyzers in DC microgrids. The topology presented in [24] proves to be an adequate choice for the charging and discharging of batteries and supercapacitors. An extendable bidirectional DC-DC converter for vehicle-to-grid (V2G) and grid-to-vehicle (G2V) applications is also proposed in [25].

In this context, this work presents a comprehensive analysis involving the properties and synthesis of single-switch non-isolated DC-DC converters with a wide conversion range derived from the six classical topologies. The main contributions of this study include the following:

− Investigating the modularity of multistage converters aiming at obtaining single-switch structures and extending the concept formerly introduced in [12];
− Applying the graft scheme in the conception of structures with distinct characteristics for high step-down and/or high step-up applications;
− Assessing the properties of derived topologies in terms of the voltage gain, current and voltage stresses, and behavior of the input and output stage currents.

This work is organized as follows. Section 2 addresses the limited capacity of the traditional buck and boost converters in achieving high step-down and high step-up, respectively. Section 3 revisits some basic concepts regarding cascaded converters, while Section 4 discusses the advantages and disadvantages of the graft scheme in generating single-switch topologies. Section 5 shows how one can obtain multistage single-switch topologies based on the six classical non-isolated DC-DC converters. Section 6 summarizes several characteristics desirable for the conception of high step-up and/or high step-down topologies, including some arrangements reported in the literature and their related applications. Section 7 concludes this study and highlights potential future work on the subject.

## 2. Limitations of Classical Non-Isolated DC-DC Converters

When galvanic isolation is not a must, non-isolated DC-DC converters can be used instead, with the consequent reduction in dimensions and increase in efficiency due to the lack of a high-frequency transformer [26]. The traditional buck and boost converters are the preliminary choices in voltage step-up or step-down, respectively, mainly owing to simplicity and low component count, although some important practical issues must be taken into account. A thorough overview of fundamentals involving basic converter topologies is presented in [27].

**Figure 1:**
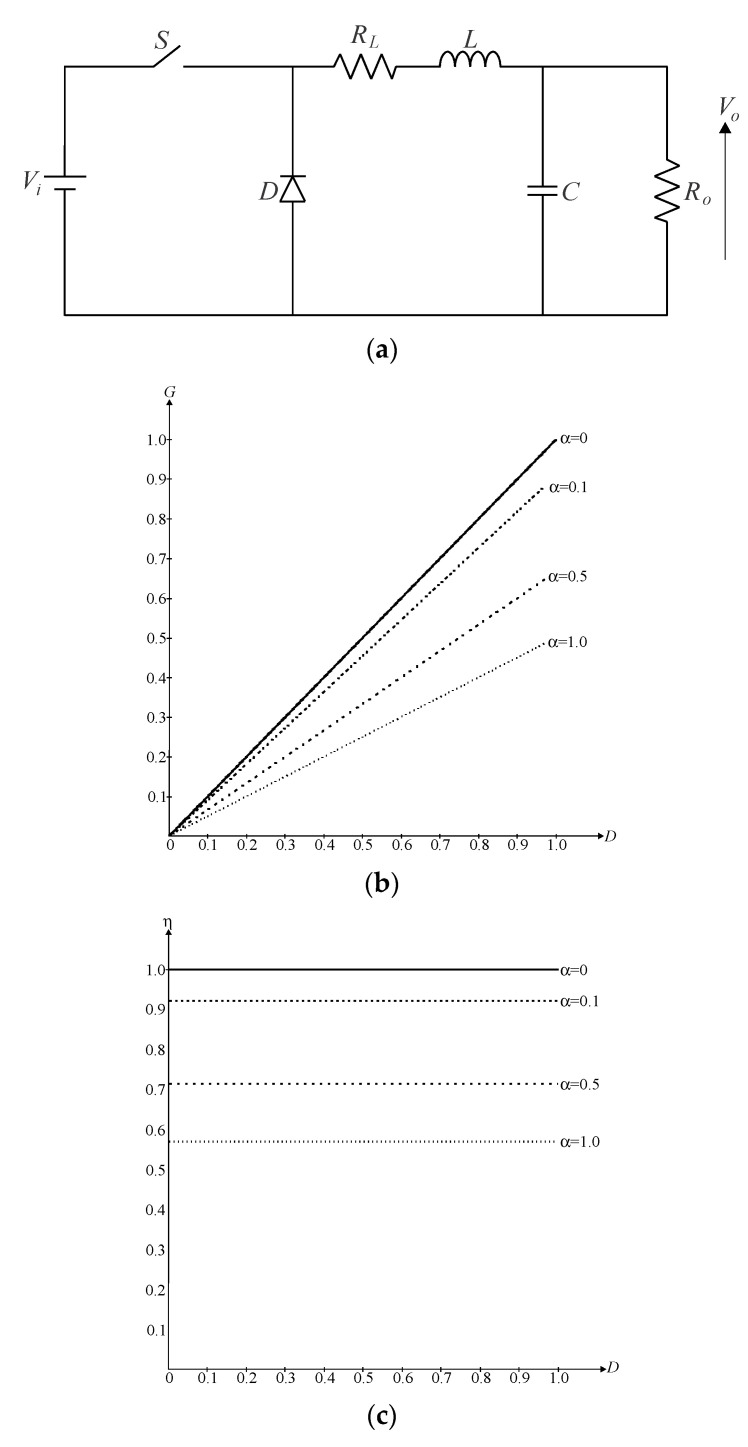
Conventional DC-DC buck converter: (**a**) power stage considering the presence of parasitic elements, (**b**) resulting voltage gain curves as a function of the duty cycle, and (**c**) efficiency curves as a function of the duty cycle.

First, let us consider the buck converter shown in Figure 1a, where the intrinsic series resistance of the filter inductor represented by *R_L_* is the only parasitic element in the circuit. Thus, one can easily demonstrate that the voltage gain *G* of the topology in continuous conduction mode (CCM) is given by (1).

(1) $$G=\frac{V_{o}}{V_{i}}=\frac{D}{1+\alpha },$$

where *V_o_* is the average output voltage, *V_i_* is the average input voltage, *D* is the duty cycle, and *α* is the ratio between *R_L_* and the load resistance *R_o_* defined as in (2).

(2) $$\alpha =\frac{R_{L}}{R_{o}}.$$

Figure 1b shows that the voltage gain is directly influenced by *R_L_*, as it is necessary to impose higher duty ratios on the active switch to obtain a given conversion ratio when compared with the ideal converter in which *α* = 0, i.e., *R_L_* = 0.

The presence of *R_L_* also affects the converter efficiency, according to Figure 1c, especially in high-power applications, since the losses increase with the square of the root-mean-square (RMS) current through the inductor, which is approximately equal to the average output current *I_o_* in the buck converter. It is also possible to demonstrate that the efficiency *η* is given by (3).

(3) $$\eta =\frac{1}{1+\alpha }.$$

Another important aspect to be observed in (1) lies in the fact that wide voltage conversion ranges are only possible when dealing with very low duty ratios, which may not be feasible in practical applications. It is worth mentioning that actual gating signals applied to active switches have finite *dv*/*dt* rates, while fast semiconductor elements such as metal–oxide–semiconductor field-effect transistors (MOSFETs) have finite turn-on and turn-off times. Moreover, obtaining very low and/or high duty ratios typically requires expensive and complex fast drivers.

**Figure 2:**
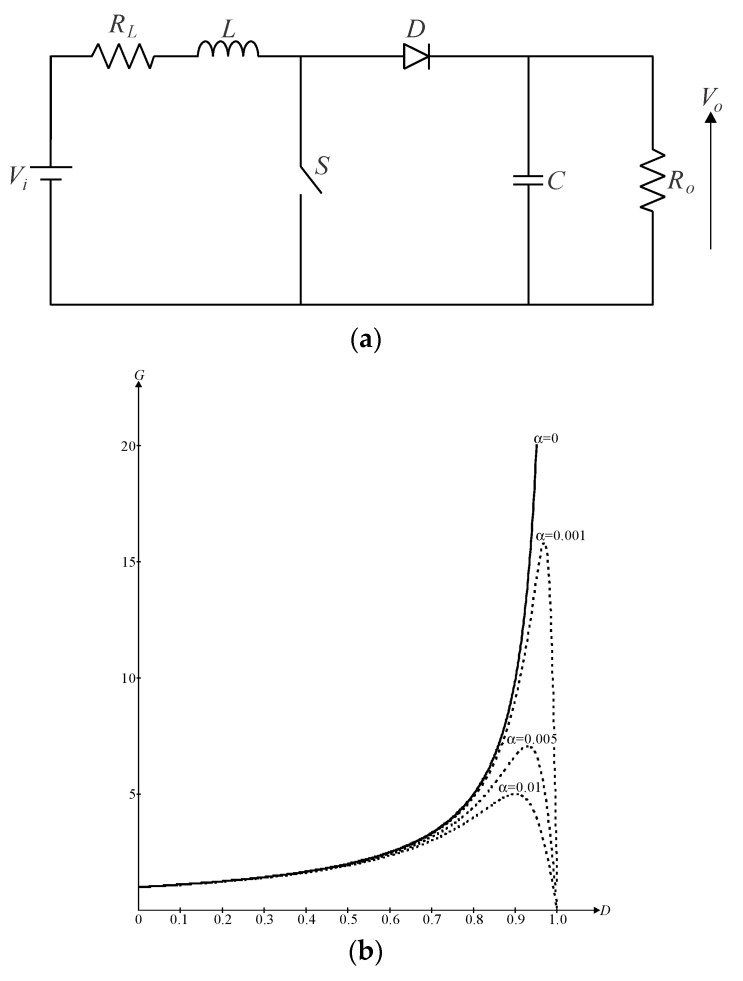

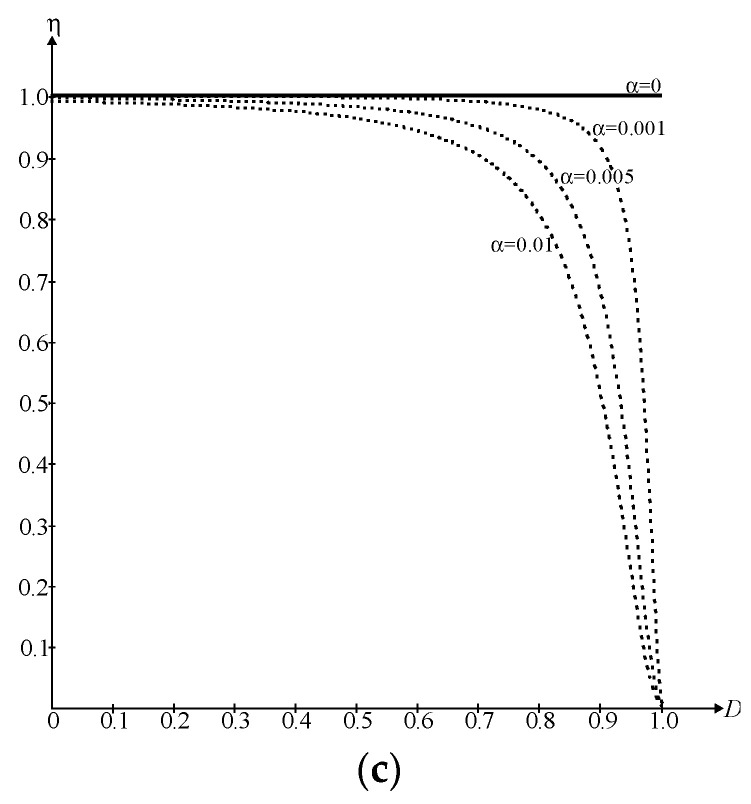
Conventional DC-DC boost converter: (**a**) power stage considering the presence of parasitic elements, (**b**) resulting voltage gain curves as a function of the duty cycle, and (**c**) efficiency curves as a function of the duty cycle.

Now, let us analyze the classical DC-DC boost topology represented in Figure 2a, where the presence of *R_L_* is also considered. The voltage gain of the converter operating in CCM can then be derived as in (4).

(4) $$G=\frac{V_{o}}{V_{i}}=\left(\frac{1}{1-D}\right)\frac{1}{\left[1+\frac{\alpha }{{\left(1-D\right)}^{2}}\right]}.$$

If the DC-DC boost converter is ideal, that is, *α* = 0, the output voltage will tend to infinity as the duty cycle tends to unity according to Figure 2b, but it would demand the use of complex and costly drive circuitry. The voltage gain, in practice, is limited to a finite value because high output voltages would demand high duty ratios, thus causing the switch to remain on for long time intervals. If the current through the diode is high, serious drawbacks regarding the reverse recovery phenomenon also exist. Now, considering *α* = 0.01 in Figure 2b, one can state that the boost converter cannot achieve high step-up, whereas the voltage gain tends to decrease at very high duty ratios owing to the voltage drop on the filter inductor.

One can also determine the theoretical efficiency of the boost converter from (5). Figure 2c shows that *R_L_* affects the converter efficiency directly. This issue becomes more evident at high power levels since the inductor losses increase with the square of the RMS input current. Other relevant factors impacting efficiency include the equivalent series resistance (ESR) of the output capacitor, reverse recovery losses, high *dv*/*dt* and *di*/*dt* rates associated with the diode, and high voltage stresses across the semiconductor elements. Therefore, it is reasonable to state that the conventional boost converter is a simple choice for applications that do not demand high step-up.

(5) $$G=\frac{V_{o}}{V_{i}}=\frac{1}{1-D}\cdot \frac{1}{\left(1+\frac{\alpha }{{\left(1-D\right)}^{2}}\right)}.$$

## 3. Cascaded Non-Isolated DC-DC Converters

**Figure 3:**
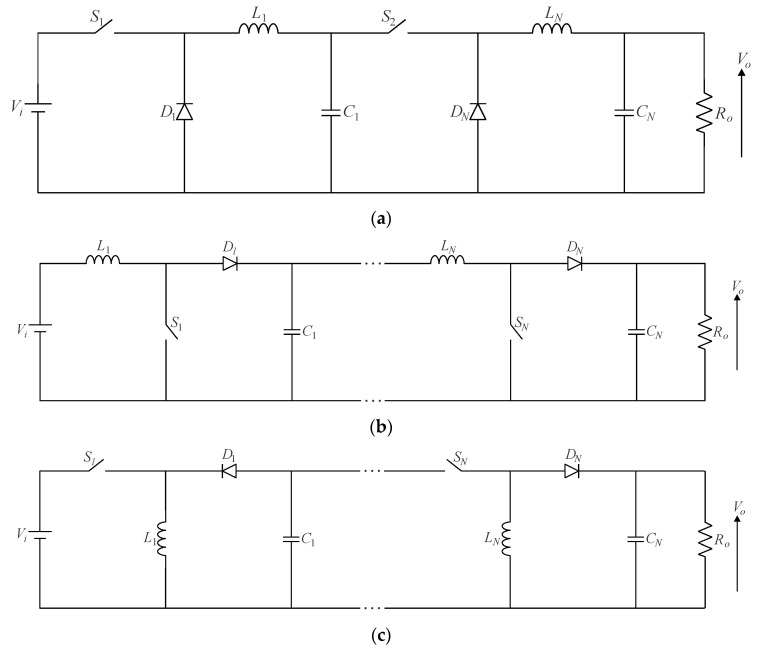
Cascaded DC-DC converters with multiple active switches: (**a**) buck converter, (**b**) boost converter, and (**c**) buck–boost converter.

Cascading power converters is a simple and straightforward approach that yields a wide conversion range, whereas one can virtually extend this concept to any topology [28,29]. To achieve high step-up and/or high step-down, the cascade connection of classical DC-DC non-isolated converters is possible, according to Figure 3. It is worth noting that in buck–boost and Ćuk converters, the output voltage polarity is opposite to that of the input voltage. This requires verifying the placement of the active switch and diode in each cascaded stage to ensure proper circuit operation. As an example, let us consider the total number of stages as *N* = 2 in Figure 3c. The active switches and diodes of the input stage and its subsequent circuit are positioned to allow current flow through them in opposite directions. This very same reasoning applies to a higher number of power stages.

The following advantages can be attributed to cascaded converters [30]:

− Inherent modularity;
− The voltage gain of the resulting association is equal to the product of the voltage gains associated with the individual stages;
− The active switches can be driven independently with distinct duty ratios;
− Additional degrees of freedom can be incorporated into the control system.
− In turn, significant drawbacks tend to exist:
− High component count, especially when many cascaded converters are used to obtain a wide conversion range;
− All active switches must be turned on simultaneously, as the gating signals must be properly synchronized;
− Reduced robustness due to the presence of several semiconductor elements, while the converter becomes more susceptible to eventual malfunctioning;
− The overall efficiency, determined by the product of the efficiencies of each stage in the cascade configuration, is significantly reduced due to energy flowing through multiple power stages;
− The control system may become significantly complex;
− The current and voltage stresses involving the semiconductor elements in the last stage may be somewhat high, thus limiting the application of cascaded converters to low power levels.

Considering that all converters in Figure 3 operate in CCM, the voltage gains of the *N*-stage cascaded buck, boost, and buck–boost converters are given by (6)–(8), respectively.

(6) $$G=\frac{V_{o}}{V_{i}}=\prod_{n=1}^{N}D_{n},$$

(7) $$G=\frac{V_{o}}{V_{i}}=\prod_{n=1}^{N}\left(\frac{1}{1-D_{n}}\right),$$

(8) $$G=\frac{V_{o}}{V_{i}}=\left\{\begin{array}{c} \prod_{n=1}^{N}\left(-\frac{D_{n}}{1-D_{n}}\right),\;\mathrm{if}\;n=1,3,5\ldots \\ \prod_{n=1}^{N}\left(+\frac{D_{n}}{1-D_{n}}\right),\;\mathrm{if}\;n=2,4,6\ldots \end{array}\right.,$$

where *D*_1_… *D_N_* are the duty ratios associated with active switches *S*_1_…*S_N_*, respectively; *N* is the number of cascaded converters; and *n* = 1, 2, … *N* corresponds to a given stage. It is also noteworthy that “+” and “−” in (8) denote that the output voltage is positive or negative, respectively.

**Figure 4:**
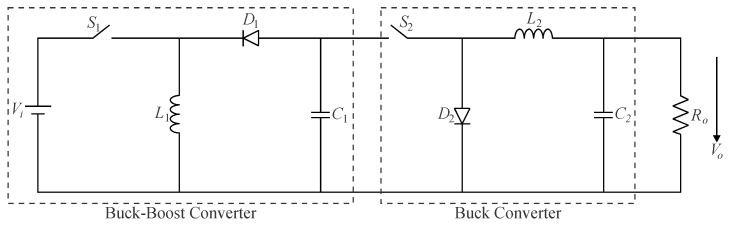
Two-switch cascaded buck–boost/buck converter.

This concept can also be applied in the cascade connection of distinct topologies, such as the two-stage association represented in Figure 4, in which a buck converter follows a buck–boost one. If both stages operate in CCM, it is easy to demonstrate that the voltage gain is calculated from (9).

(9) $$G=\frac{V_{o}}{V_{i}}=\left(-\frac{D_{1}}{1-D_{1}}\right)D_{2},$$

where *D*_1_ and *D*_2_ are the duty cycles of switches *S*_1_ and *S*_2_, respectively. Furthermore, it is observed that the output voltage polarity is opposite to that of the input voltage owing to modifications in the positions of the active switch and the diode in the output stage compared to the traditional buck topology.

Even though numerous configurations can be obtained in practice, the use of many active switches may lead to increased cost and complexity regarding the control system and/or drive circuitry. It is then desirable to integrate stages so that single-switch topologies can be derived instead using the graft scheme described as follows.

## 4. Graft Scheme Applied in the Conception of Single-Switch Quadratic DC-DC Converters

**Figure 5:**
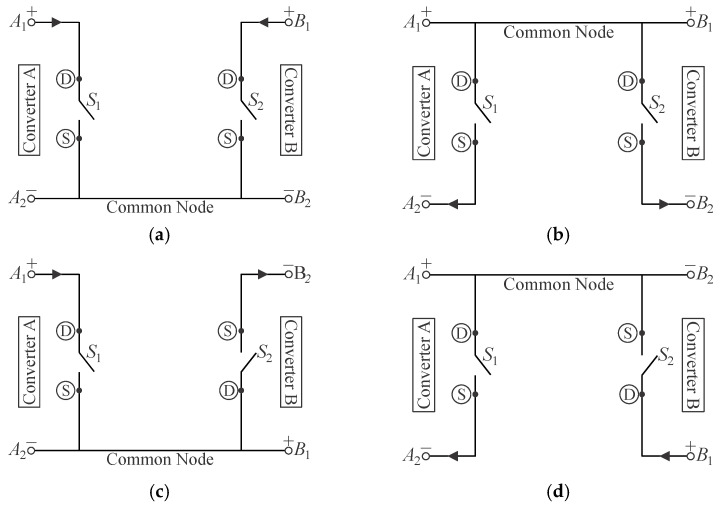
Possible configurations for two active switches connected to a common point: (**a**) configuration I—source–source connection, (**b**) configuration II—drain–drain connection, (**c**) configuration III—source–drain connection, and (**d**) configuration IV—drain–source connection.

One can properly integrate two cascaded converters relying on one active switch each using the so-called graft technique, considering the four possible arrangements described in [13,27] and shown in Figure 5. This concept enables the derivation of small-signal models for single-stage topologies based on basic converter units [31] and assessing their operation in discontinuous conduction mode (DCM) [32].

**Figure 6:**
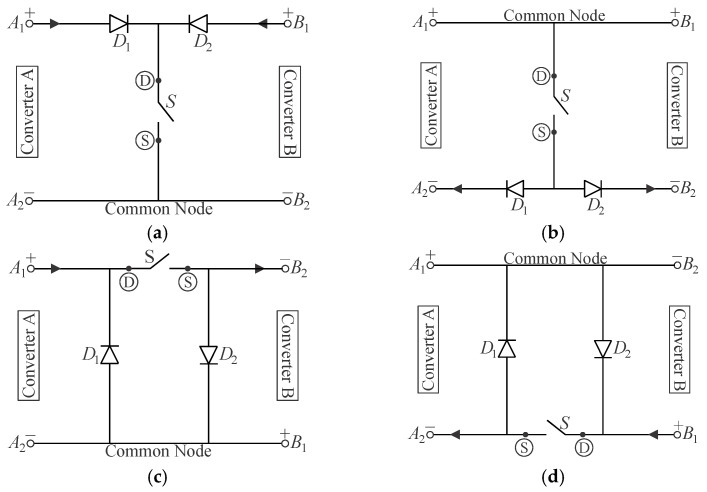
Resulting single-switch configurations for the integration of cascaded converters: (**a**) configuration I, (**b**) configuration II, (**c**) configuration III, and (**d**) configuration IV.

Configurations I and II correspond to common source-source and common drain-drain connections, respectively, while configurations III and IV consist of common drain-source and common source-drain connections, respectively. According to [13], the two active switches in each possible combination can be replaced with an arrangement composed of one single active switch and two diodes, yielding the corresponding circuits represented in Figure 6, where “D” and “S” stand for the drain and source terminals of a MOSFET, respectively.

**Figure 7:**
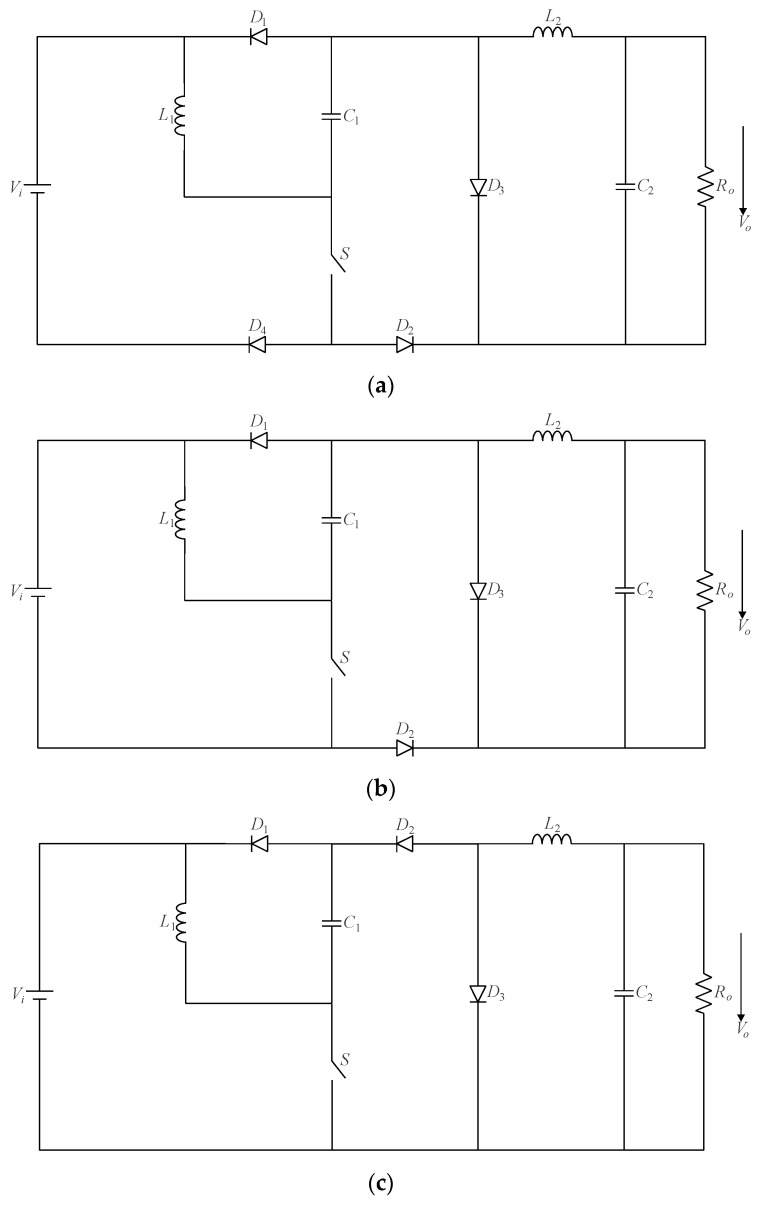
Single-switch cascaded buck–boost/buck converter: (**a**) configuration with two additional diodes, (**b**) topology with a single diode, and (**c**) resulting modified topology.

Now, let us consider the cascaded buck–boost/buck converter shown in Figure 4 as an example. Analyzing the circuit, one can identify the existence of configuration III according to Figure 5c. Thus, it is possible to obtain the resulting single-switch converter in Figure 7a from Figure 6c, where two diodes are connected to the active switch. However, since diode *D*_4_ is responsible for ensuring the current flow in a single direction while always remaining forward-biased during the converter operation, one can replace such an element with a short circuit, as shown in Figure 7b. Another topological variation can be derived in Figure 7c, where the position of diode *D*_2_ is modified in the circuit.

Applying the volt-second balance to Figure 7, one can obtain the voltage gain of the converter operating in CCM as in (10).

(10) $$G=\frac{V_{o}}{V_{i}}=-\frac{D^{2}}{1-D}.$$

Despite the reduced cost and increased robustness and reliability associated with single-switch cascaded topologies, there are still some drawbacks [33]. Configurations I and II cause increased current stresses on the switch, while configurations III and IV yield increased voltage stresses on the switch. In configurations I and II, the current stress equals the sum of the currents from both stages. Conversely, in configurations III and IV, it corresponds to the higher of the two currents from the two stages.

## 5. Deriving Multistage Single-Switch Non-Isolated DC-DC Converters

Alternate topologies with different voltage gain characteristics can be synthesized by cascading basic converters with each other [34]. In addition, it has been demonstrated in [12] that single-switch converters with non-standard conversion ratios, referred to as quadratic converters, can be derived. Even though such structures can achieve wide voltage conversion ratios, they are not widely used for being generally less efficient than other topologies, although they can be useful for some applications [35].

**Figure 8:**
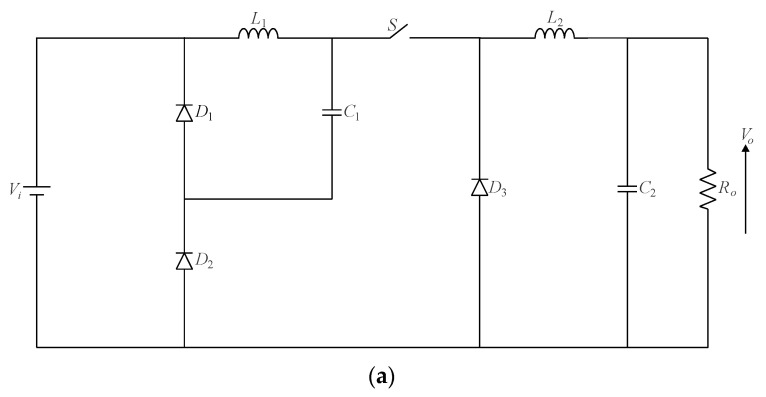

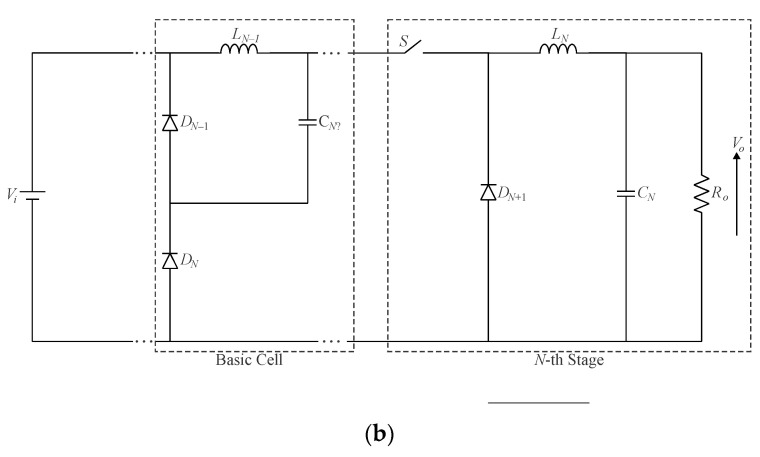
Single-switch quadratic buck converter: (**a**) original topology proposed in [12] and (**b**) converter modularity.

As previously mentioned, the graft scheme proposed in [13] allows for deriving single-switch converters as long as two or more switches share a common connection node. In this sense, it is possible to obtain multistage single-switch non-isolated DC-DC converters based on cascade stages in terms of a modular approach, which has not been presented in the literature before. Let us start with the quadratic buck converter formerly introduced in [12] and shown in Figure 8. Since it consists of the association of two buck stages, one can obtain a single-switch version of the topology by considering the active switches connected to each other in the form of configuration IV, according to Figure 5d. Adopting the resulting arrangement shown in Figure 6d and rearranging the position of power stage elements yields the circuit in Figure 8a, which has a fourth-order characteristic [36]. It is also possible to obtain a modular approach based on a single active switch from the same reasoning, resulting in the multistage converter shown in Figure 8b. This principle was applied in the conception of a cubic buck converter in [37].

**Figure 9:**
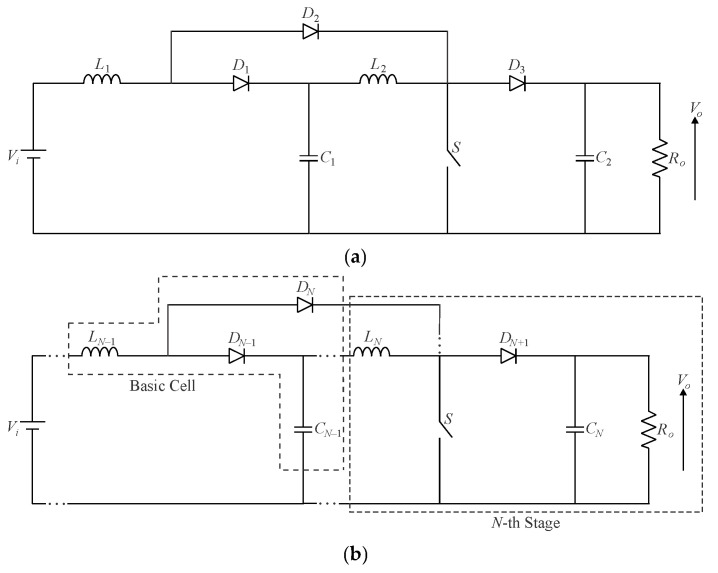
Single-switch quadratic boost converter: (**a**) original topology proposed in [12] and (**b**) converter modularity.

The quadratic boost converter shown in Figure 9a is perhaps one of the first non-isolated high step-up DC-DC converters reported in the literature [12]. Despite the output voltage increasing in a quadratic ratio, the voltage stresses on the active switch *S* and the output diode *D*_3_ remain equal to the output voltage, as observed in the traditional boost converter. Moreover, since this single-switch topology is obtained from configuration I, as shown in Figure 5a and Figure 6a, the active switch is subjected to a higher RMS current, which leads to higher conduction losses. These two aforementioned issues may restrict the converter to low-power, low-output-voltage applications. It is also worth mentioning that one can derive a multistage single-switch boost converter using the graft scheme in terms of Figure 9b.

**Figure 10:**
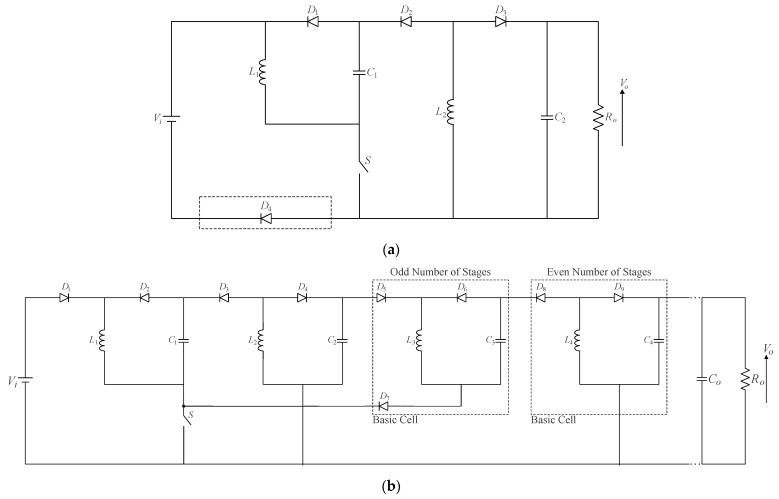
Single-switch quadratic buck–boost converter: (**a**) original topology proposed in [12] and (**b**) converter modularity.

The quadratic buck–boost converter shown in Figure 10a was formerly proposed in [12]. The resulting voltage gain in CCM corresponds to the products of the voltage gains of two conventional buck–boost converters. In other words, the topology can provide high step-down and high step-up if *D* < 0.5 and *D* > 0.5. However, if diode *D*_4_ is replaced with a short circuit, as in [12], the converter will only be capable of operating in step-down mode. Incorporating such a diode into the circuit shown in Figure 10b allows for obtaining a multistage structure that can operate in either mode. It is also noteworthy that different basic cells are utilized in deriving such topology, depending on whether the number of cascaded stages is odd or even. An application of the quadratic buck–boost converter operating as an LED driver capable of providing a high input power factor is presented in [38]. The graft scheme was also employed to derive a cubic buck–boost topology with a high step-up capability in [39].

An important issue is that only topologies based on the basic second-order converters were addressed in [12], while the quadratic Ćuk, SEPIC, and Zeta topologies remained unexplored at that time. Let us recall that the Ćuk converter is a fourth-order system in which the boost and buck converters are arranged as the input and output stages, respectively [40]. Another topological variation referred to as the SEPIC converter was introduced in [41], whose input and output stages consist of the boost and buck–boost converters, respectively. The last classical non-isolated DC-DC converter, which is referred to as dual SEPIC or Zeta converter, relies on the association of the buck–boost and buck converters [42].

Other quadratic step-up/step-down DC-DC topologies based on the classical Ćuk, SEPIC, and Zeta converters can be derived using the graft technique. While they can operate over the entire duty cycle range 0 ≤ *D* ≤ 1, their practical application may be restricted owing to the high voltage stress on the active switch, high component count, and significant complexity involved in implementing the control system owing to the high system order. Furthermore, the need for four inductors may lead to increased size, weight, and volume.

**Figure 11:**
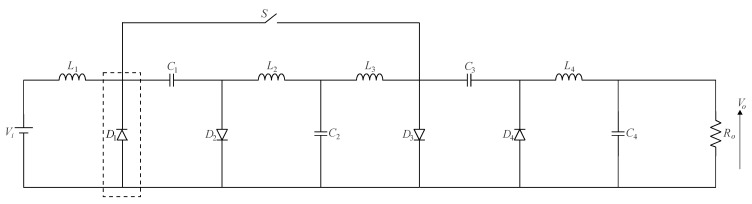
Single-switch quadratic Ćuk converter.

**Figure 12:**
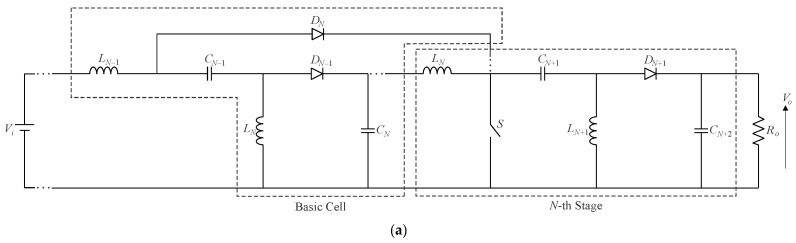

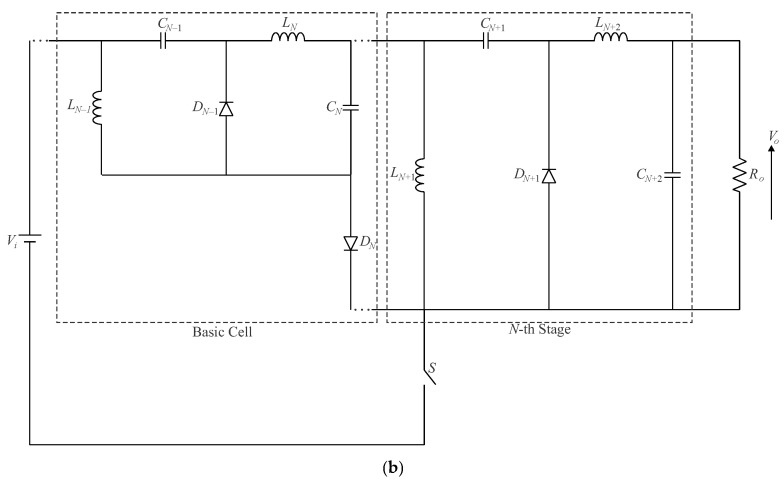
Single-switch multistage DC-DC topologies: (**a**) SEPIC converter and (**b**) Zeta converter.

Figure 11 shows the quadratic Ćuk converter, which, like the quadratic buck–boost converter, generates a positive output voltage. Owing to the position of the active switch in the circuit, a priori, it is not possible to arrange it in a single-switch modular form aiming to extend the voltage gain. Even though it presents a high component count, a modified quadratic Ćuk topology based on three inductors and three capacitors was proposed in [43]. In turn, the modular versions of the SEPIC and Zeta converters are presented in Figure 12a,b, respectively. It is necessary to cascade the basic cells represented in the dashed boxes to extend the conversion ratio, as well as to maintain the *N*-th stage associated with the output stage in any resulting converter. A quadratic AC-DC quadratic SEPIC converter employed as an LED driver was proposed in [44], resulting in a high input power factor and high efficiency over a wide load range while considering a moderate voltage gain. It is also noteworthy that the quadratic Zeta converter shown in Figure 12b remains unexplored thus far.

## 6. Properties and Synthesis of Two-Stage Single-Switch Non-Isolated DC-DC Converters

The graft scheme is a versatile approach to derive numerous DC-DC converter structures beyond those outlined in [12]. By combining the classical buck, boost, buck–boost, Ćuk, SEPIC, and Zeta converters, it is possible to derive 36 two-stage, single-switch topologies with the most diverse characteristics [45]. In this sense, this section presents a brief discussion of non-isolated DC-DC converters based on the cascaded association of two distinct stages, which can be referred to as hybrid quadratic topologies.

The AC-DC SEPIC/buck–boost converter was proposed in [46] as an offline LED driver that does not rely on electrolytic capacitors. The input stage can operate in DCM and provide power-factor correction (PFC) owing to the voltage follower characteristic of the SEPIC converter. The buck stage operates in CCM as it is responsible for power flow control and LED dimming.

The SEPIC–buck converter is also a compelling option for LED-based lighting applications because the currents through both the input and output stages are non-pulsating, resulting in reduced EMI levels [47]. Similar to [46], the input stage operates in DCM, minimizing the size of the filter inductor and eliminating the need for a control loop for the input current. The output buck stage operates in CCM, aiming to control the current through the LEDs. However, this topology is only adequate for high step-down applications because it only operates in step-up mode for *D* > 0.618. In other words, it means that the circuit is not competitive even when compared with the classical boost converter because it will require a higher duty ratio to provide the same voltage gain.

The SEPIC–Ćuk converter presented in [48] also benefits from non-pulsating currents in both stages. Even though it can achieve high step-up and/or high step-down, a significant drawback lies in high component count, especially because it requires four filter inductors that may drastically impact power density. In turn, even though the boost–Ćuk converter described in [49] relies on three filter inductors instead, it is better recommended for high step-up applications only. This is because the operation in step-down mode occurs only when *D* < 0.382, whereas the converter would require very low duty ratios to provide a high step-down.

**Figure 13:**
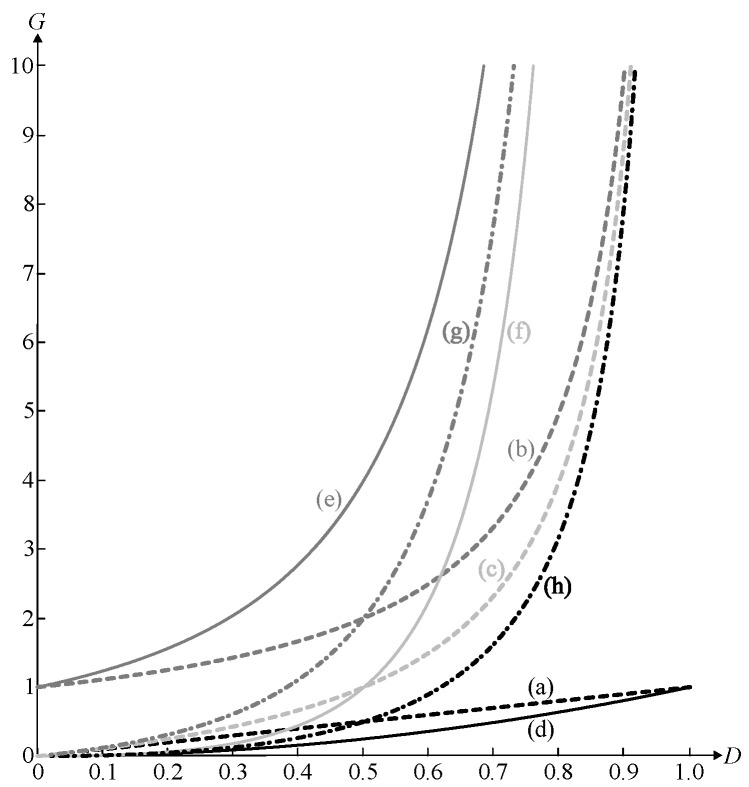
Voltage gains of single-switch conventional and quadratic non-isolated DC-DC converters operating in CCM as a function of the duty cycle: (a) low step-down, (b) low step-up, (c) low step-up/low step-down, (d) high step-down, (e) high step-up, (f) high step-up/high step-down, (g) high step-up/low step-down, and (h) low step-up/high step-down.

Considering that the voltage gain varies as a function of the duty cycle, as shown in Figure 13, it is reasonable to classify the conventional and quadratic single-switch non-isolated DC-DC converters operating into CCM into six types:

(i) Topologies adequate for low step-down (a), low step-up (b), or low step-up/low step-down applications (c), this being the case of the classical buck, boost, and buck–boost converters, respectively;

(ii) Topologies adequate for high step-down applications (d);

(iii) Topologies adequate for high step-up applications (e);

(iv) Topologies adequate for high step-up/high step-down applications (f);

(v) Topologies adequate for high step-up/low step-down applications (g);

(vi) Topologies adequate for low step-up/high step-down applications (h).

While some topologies have been assessed in the literature, many structures remain unexplored thus far. In this sense, Table 1, Table 2, Table 3, Table 4, Table 5 and Table 6 summarize some important characteristics of all possible combinations, which can be useful for selecting a given topology. For instance, among applications that require a high step-up stage, grid-connected photovoltaic (PV) systems rely on a front-end DC-DC stage for stepping up the low voltage across the modules and supplying a cascaded inverter [50]. The quadratic boost and boost–Ćuk converters could also be adequate choices for this purpose, considering proper tradeoffs among the voltage gain, stresses on semiconductors, and behavior of the currents through the input and output stages. In turn, applications involving battery charging would benefit from the non-pulsating currents of the SEPIC–buck converter operating in CCM, whereas the topology can be connected to the AC grid and achieve input PFC.

## 7. Conclusions

This work has presented the properties and synthesis of single-switch quadratic non-isolated DC-DC converters employing the graft scheme. This principle consists of a useful approach for generating novel power converter topologies while numerous arrangements exist. Combining the six basic non-isolated topologies yields 36 structures with distinct characteristics. Of course, not all of them are feasible for practical applications, whereas it is necessary to consider tradeoffs among the voltage gain, stresses on semiconductors, and component count, among other aspects.

Cascaded converters comprising multiple active switches introduce more flexibility into the control system, whereas the switches can operate independently with distinct duty ratios. In turn, significant drawbacks may include the need for isolated gate drivers and the fact that all switches must be turned on simultaneously, thus requiring complex and costly circuits. In turn, one can derive single-switch counterparts from the graft scheme, but at the cost of additional current or voltage stresses on the switch.

The voltage stress across the semiconductor elements is a key issue in either multiple-switch cascaded or single-switch quadratic DC-DC converters. It is worth mentioning that cost increases significantly as the maximum voltage ratings regarding semiconductors also do, consequently implying increased conduction losses and affecting the converter efficiency.

It is reasonable to state that quadratic converters are suitable for applications with moderately wide conversion ranges, where classical single-stage non-isolated DC-DC converters may be inadequate. Furthermore, such topologies may be restricted to low power levels and low input and/or output voltages so that efficiency is not seriously affected by high losses due to high component count and/or additional current and voltage stresses.

Future work includes investigating proper techniques to reduce voltage stresses across semiconductor elements in single-switch quadratic DC-DC converters, as well as application-specific design considerations for such topologies in particular.

## Figures and Tables

**Table 1:** Single-switch quadratic topologies derived from the buck converter.

Characteristic	Buck/Buck [12]	Buck/Boost	Buck/Buck–Boost	Buck/Ćuk	Buck/SEPIC	Buck/Zeta
Configuration	IV	IV	IV	IV	IV	IV
Voltage gain	$D^{2}$ (d)	$\frac{D}{1-D}$ (c)	$-\frac{D^{2}}{1-D}$ (h)	$-\frac{D^{2}}{1-D}$ (h)	$\frac{D^{2}}{1-D}$ (h)	$\frac{D^{2}}{1-D}$ (h)
Input current	Pulsating	Pulsating	Pulsating	Pulsating	Pulsating	Pulsating
Output stage current	Non-Pulsating	Pulsating	Pulsating	Non-Pulsating	Pulsating	Non-Pulsating
Switch Additional Stresses	Voltage	Voltage	Voltage	Voltage	Voltage	Voltage
Components (*S*/*D*/*L*/*C*)	1/3/2/2	1/3/2/2	1/3/2/2	1/3/3/3	1/3/3/3	1/3/3/3
Applications	High step-down	Low step-up/low step-down	Low step-up/high step-down	Low step-up/high step-down	Low step-up/high step-down	Low step-up/high step-down

**Table 2:** Single-switch quadratic topologies derived from the boost converter.

Characteristic	Boost/Buck	Boost/Boost [12]	Boost/Buck–Boost	Boost/Ćuk [49]	Boost/SEPIC	Boost/Zeta
Configuration	I	I	I	I	I	I
Voltage gain	$\frac{D}{1-D}$ (c)	${\left(\frac{1}{1-D}\right)}^{2}$ (e)	$-\frac{D}{{\left(1-D\right)}^{2}}$ (g)	$-\frac{D}{{\left(1-D\right)}^{2}}$ (g)	$\frac{D}{{\left(1-D\right)}^{2}}$ (g)	$\frac{D}{{\left(1-D\right)}^{2}}$ (g)
Input current	Non-Pulsating	Non-Pulsating	Non-Pulsating	Non-Pulsating	Non-Pulsating	Non-Pulsating
Output stage current	Non-Pulsating	Pulsating	Pulsating	Non-Pulsating	Pulsating	Non-Pulsating
Switch Additional Stresses	Current	Current	Current	Current	Current	Current
Components (*S*/*D*/*L*/*C*)	1/3/2/2	1/3/2/2	1/3/2/2	1/3/3/3	1/3/3/3	1/3/3/3
Applications	Low step-up/low step-down	High step-up	High step-up/low step-down	High step-up/low step-down	High step-up/low step-down	High step-up/low step-down

**Table 3:** Single-switch quadratic topologies derived from the buck–boost converter.

Characteristic	Buck–Boost/Buck	Buck–Boost/Boost	Buck–Boost/Buck–Boost [12]	Buck–Boost/Ćuk	Buck–Boost/SEPIC	Buck–Boost/Zeta
Configuration	III	III	III	III	III	III
Voltage gain	$-\frac{D^{2}}{1-D}$ (h)	$-\frac{D}{{\left(1-D\right)}^{2}}$ (g)	${\left(\frac{D}{1-D}\right)}^{2}$ (f)	${\left(\frac{D}{1-D}\right)}^{2}$ (f)	$-{\left(\frac{D}{1-D}\right)}^{2}$ (f)	$-{\left(\frac{D}{1-D}\right)}^{2}$ (f)
Input current	Pulsating	Pulsating	Pulsating	Pulsating	Pulsating	Pulsating
Output stage current	Non-Pulsating	Pulsating	Pulsating	Non-Pulsating	Pulsating	Non-Pulsating
Switch Additional Stresses	Voltage	Voltage	Voltage	Voltage	Voltage	Voltage
Components (*S*/*D*/*L*/*C*)	1/3/2/2	1/3/2/2	1/4/2/2	1/4/3/3	1/4/3/3	1/4/3/3
Applications	Low step-up/high step-down	High step-up/low step-down	High step-up/high step-down	High step-up/high step-down	High step-up/high step-down	High step-up/high step-down

**Table 4:** Single-switch quadratic topologies derived from the Ćuk converter.

Characteristic	Ćuk/Buck	Ćuk/Boost	Ćuk/Buck–Boost	Ćuk/Ćuk	Ćuk/SEPIC	Ćuk/Zeta
Configuration	I	I	I	I	I	I
Voltage gain	$-\frac{D^{2}}{1-D}$ (h)	$-\frac{D}{{\left(1-D\right)}^{2}}$ (g)	${\left(\frac{D}{1-D}\right)}^{2}$ (f)	${\left(\frac{D}{1-D}\right)}^{2}$ (f)	$-{\left(\frac{D}{1-D}\right)}^{2}$ (f)	$-{\left(\frac{D}{1-D}\right)}^{2}$ (f)
Input current	Non-Pulsating	Non-Pulsating	Non-Pulsating	Non-Pulsating	Non-Pulsating	Non-Pulsating
Output stage current	Non-Pulsating	Pulsating	Pulsating	Non-Pulsating	Pulsating	Non-Pulsating
Switch Additional Stresses	Current	Current	Current	Current	Current	Current
Components (*S*/*D*/*L*/*C*)	1/3/3/3	1/3/3/3	1/4/3/3	1/4/4/4	1/4/4/4	1/4/4/4
Applications	Low step-up/high step-down	High step-up/low step-down	High step-up/high step-down	High step-up/high step-down	High step-up/high step-down	High step-up/high step-down

**Table 5:** Single-switch quadratic topologies derived from the SEPIC converter.

Characteristic	SEPIC/Buck [47]	SEPIC/Boost	SEPIC/Buck–Boost[46]	SEPIC/Ćuk [48]	SEPIC/SEPIC	SEPIC/Zeta
Configuration	I	I	I	I	I	I
Voltage gain	$\frac{D^{2}}{1-D}$ (h)	$\frac{D}{{\left(1-D\right)}^{2}}$ (g)	${\left(\frac{D}{1-D}\right)}^{2}$ (f)	${\left(\frac{D}{1-D}\right)}^{2}$ (f)	${\left(\frac{D}{1-D}\right)}^{2}$ (f)	${\left(\frac{D}{1-D}\right)}^{2}$ (f)
Input current	Non-Pulsating	Non-Pulsating	Non-Pulsating	Non-Pulsating	Non-Pulsating	Non-Pulsating
Output stage current	Non-Pulsating	Pulsating	Pulsating	Non-Pulsating	Pulsating	Non-Pulsating
Switch Additional Stresses	Current	Current	Current	Current	Current	Current
Components (*S*/*D*/*L*/*C*)	1/3/3/3	1/3/3/3	1/4/3/3	1/4/4/4	1/4/4/4	1/4/4/4
Applications	Low step-up/high step-down	High step-up/low step-down	High step-up/high step-down	High step-up/high step-down	High step-up/high step-down	High step-up/high step-down

**Table 6:** Single-switch quadratic topologies derived from the Zeta converter.

Characteristic	Zeta/Buck [47]	Zeta/Boost	Zeta/Buck–Boost [46]	Zeta/Ćuk [48]	Zeta/SEPIC	Zeta/Zeta
Configuration	IV	IV	IV	IV	IV	IV
Voltage gain	$\frac{D^{2}}{1-D}$ (h)	$\frac{D}{{\left(1-D\right)}^{2}}$ (g)	${\left(\frac{D}{1-D}\right)}^{2}$ (f)	${\left(\frac{D}{1-D}\right)}^{2}$ (f)	${\left(\frac{D}{1-D}\right)}^{2}$ (f)	${\left(\frac{D}{1-D}\right)}^{2}$ (f)
Input current	Pulsating	Pulsating	Pulsating	Pulsating	Pulsating	Pulsating
Output stage current	Non-Pulsating	Pulsating	Pulsating	Non-Pulsating	Pulsating	Non-Pulsating
Switch Additional Stresses	Voltage	Voltage	Voltage	Voltage	Voltage	Voltage
Components (*S*/*D*/*L*/*C*)	1/3/3/3	1/3/3/3	1/4/3/3	1/4/4/4	1/4/4/4	1/4/4/4
Applications	Low step-up/high step-down	High step-up/low step-down	High step-up/high step-down	High step-up/high step-down	High step-up/high step-down	High step-up/high step-down

## Data Availability

Data are available upon request from the authors.
